# Thermogravimetric analysis of the pyrolysis and combustion kinetics of surface dead combustibles in the Daxing’an Mountains

**DOI:** 10.1371/journal.pone.0260790

**Published:** 2021-12-02

**Authors:** Yang Shu, Jinqi Zhang, Wei Li, Pengwu Zhao, Qiyue Zhang, Mei Zhou

**Affiliations:** 1 Forestry College of Inner Mongolia Agricultural University, Hohhot, China; 2 National Orientation Observation and Research Station of Saihanwula Forest Ecosystem in Inner Mongolia, Chifeng, China; Government College University Faisalabad, PAKISTAN

## Abstract

In boreal regions, the frequency of forest fires is increasing. In this study, thermogravimetric analysis was used to analyze the pyrolysis kinetics of dead surface combustibles in different forest types within the Daxing’an Mountains, China. The results show that the combustible material load of forest types, the Larix forest (LG) is relatively high. Base on the E of kinetic parameters, the LG, and Quercus forest (QM) forest types had relatively high combustibility values and comprehensive combustibility values for 1-, 10-, and 100-h time lags. According to the obtained P values, the pyrolysis of dead surface fuels with 1-, 10-, and 100-h time lags is relatively difficult in the Larix / Betula mixed forest (L-B) and QM forest types. Therefore, mixed forests of the LG, L-B, and QM tree species can be established as fire-resistant forests to establish a fire barrier, reduce the combustibility of forest stands, and reduce the possibility of forest fires.

## Introduction

Forest fires not only affect trees, soil, microorganisms, and wild animals living in the forest, they also influence landscape structure, the Earth’s carbon balance, global climate change, and biodiversity [1]. The Daxing’an Mountains are a forested area that experiences the largest area and most serious forest fires in China [2]. In recent years, the global greenhouse effect and El Niño phenomenon have significantly increased the winter temperature in the Daxing’an Mountains area. Spring and autumn are dry and rainless. In spring, the temperature rises quickly and is often accompanied by windy weather. Tall and dry trees in the forest cause lightning fires in the summer, and the danger of forest fires is increasing year by year [3]. Due to the unique climate and geographical location of the Daxing’an Mountains, the dead ground cover is thick and decomposes slowly, and a large amount of forest combustibles accumulate on the forest floor [4]. Surface dead combustibles are an important fuel for surface fires, which are the dominant type of fire in the Daxing’an Mountains area.

Thermogravimetric analysis (TGA) is commonly used to study the pyrolysis mechanisms of compounds. TGA allows the study of thermal stability and the thermal decomposition mechanism and can provide thermodynamic data and kinetic parameters of the decomposition process. Z B Laougé et al used TGA to investigate the thermal decomposition of Sida with the goal of using it as an energy source. Based on the kinetic and thermodynamic parameters obtained using the Kissinger–Akahira–Sunose (KAS) and Flynn–Wall–Ozawa (FWO) methods, the authors determined that Sida is a an important biological raw material for energy production [5].

The pyrolytic and combustion behaviors of various materials including sawdust [6, 7], elephant grass, rice husk [8], tobacco waste [9], plum stone waste [10], red pepper waste [11], soybean straw [12]; *Pongamia pinnata* [13], cassava bagasse [14], para grass [15], camel grass [16], *Prosopis juliflora* [17], chestnut shells [18], *Saccharum ravannae* L. [19], *Chara vulgaris* [20], *Spirogyra crassa* [21], and banana peel [22] have been investigated. However, relatively few studies have examined the pyrolysis kinetics of combustibles using TGA. The thermal decomposition of combustibles is the initial stage of the forest fire process. This step provides the combustible fuel that triggers the fire and facilitates the subsequent spread of the fire [23]. Fire fronts insides forests spread rapidly through surface litter, which typically contains broken pieces of dead and live bark, twigs, and leaves and serves as a fuel [24]. Wang et al used TGA to study the humus in forests of *Larix gmelinii* in the Daxing’an Mountains. They found that the pyrolysis mass loss of humus in the air is the large, and pyrolysis slows as the humus particle size increases [25]. Dimitrakopoulos et al analyzed the pyrolysis kinetics of 12 biomass samples and found that TGA is a reliable method for evaluating combustibility [26]. Jeguirim et al analyzed the pyrolysis kinetics of *Arundo donax* L. and found that increasing the heating rate slightly slowed the decomposition process and increased the rate of mass loss of hemicellulose and cellulose decomposition [27].

The pyrolysis and ignition processes of fire combustibles have become a focus of research in fire science. Niu et al used the Kissinger equation to explain the effect of the heating rate on the weight-loss curve and then predicted the peak temperature at different heating rates [28]. The results suggested that pine branches burn at high intensity but do not have a strong ability to maintain combustion. Shi studied the pyrolysis of combustible materials and proposed a multi-component kinetic model for biomass pyrolysis [29]. The pyrolysis parameters estimated from the data obtained for rosewood also produced a good fit for the pyrolysis of Chinese fir and *Fraxinus mandshurica*. Cardoso et al studied the thermal decomposition of tobacco leaf residue and sorghum bagasse under non-isothermal conditions using the Ozawa and Starink models [30]. The activation energy obtained using the single-step reaction model was between the lowest and highest values produced by the independent parallel reaction model. Damartzis et al evaluated the pyrolysis of *Cynara carduculus* stems and leaves using independent parallel reaction models [31]. The KAS and OFW models were applied to evaluate the pyrolysis kinetic parameters, and the independent parallel reaction model works best. Leoni et al studied the kinetic processes of *Pinus sylvestris* (commonly called needles) drying and thermal degradation [32]. They found that the thermal degradation of pine needles occurred in the range of 200°C–550°C, and the thermal degradation consisted of two exothermic reactions: the oxidation of evolved gases and char combustion.

Our study chooses the Coats-Redfern method with a single heating rate curve, which has the advantage of being able to estimate activation under the condition of unknown mechanism function. The widely used Coat-Redfern method can obtain more accurate pre-reference factors than other integration methods of the same type, and the accuracy of the pre-reference factors can be improved by adjusting the parameters of the Coats-Redfern temperature approximation formula. Therefore, it is feasible to use the Coats-Redfern integration method to describe the pyrolysis behavior of samples in air atmosphere [33].

In summary, it is feasible to study the combustibility and pyrolysis kinetics of forest combustibles using TGA. Understanding the combustibility and pyrolysis kinetics of forest combustibles is important for managing forest combustibles and predicting forest fires and their spread. To better predict fire propagation in the Daxing’an Mountains area of China, it is critical to understand the thermal degradation characteristics of different forest litter fuel materials. Therefore, TGA was applied in this study to analyze the pyrolysis kinetics of dead surface combustibles in different forest types in the Daxing’an Mountains area. The potential combustibility of different forest types and the response mechanisms of dead surface combustibles to forest fires were evaluated. Finally, the obtained data were used to establish a database of combustible ignition characteristics in Inner Mongolia Daxing’an Mountains. The findings provide basic data for forest fire management, including forest fuel management, the construction of fire barriers, and fire risk zoning in the Daxing’an Mountains area of Inner Mongolia.

## Materials and methods

### Research site

**Fig 1:**
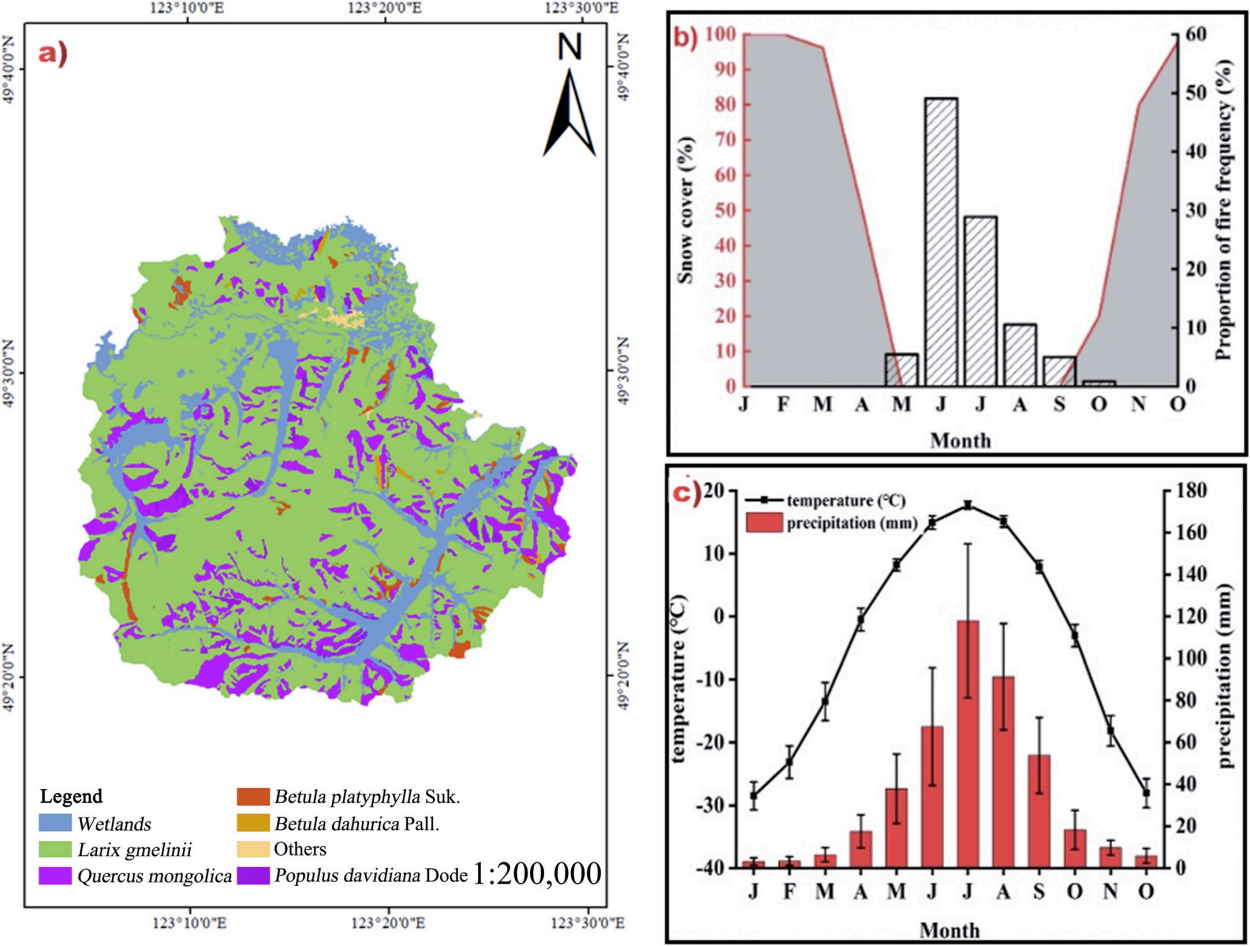
Basic information of the study area.

The research site was located in the Bila River National Nature Reserve of the Inner Mongolia Forest Industry Group (Fig 1). The geographic coordinates of the research site are: 121°24′31.03″–123°31′44.21″E, 49°31′01.92″–49°33′09.45″N. This area has a humid and semi-humid continental monsoon climate. The study area is in the mid-temperate zone and has an average annual temperature of −1.1°C. The average annual rainfall is 479.4 mm, and the rainfall period is primarily from July to August, accounting for approximately 50%–60% of total annual precipitation. The annual average temperature is below 0°C. The extreme maximum and minimum temperatures are 35.4°C and −46.0°C, respectively. The vegetation in this area is mainly composed of Siberian vegetation flora, Mongolian flora, and East Asian flora. Coniferous forests and coniferous broad-leaved mixed forests are the main forest types. As shown in Fig 1, the main tree species are *Larix sylvestris*, *Pinus sylvestris*, white birch (*Betula platyphylla Suk*.), Black birch (*Betula dahurica Pall*.), Mongolian oak, and aspen.

We selected five forest types for study: *Larix* / *Betula* mixed forest (L-B), *Betul* forest (BP), *Larix* forest (LG), *Populus* / *Betula* mixed forest (P-B), and *Quercus* forest (QM). As shown in Fig 1, the selected study site was located far from the river with a suitable slope and little human interference. A representative standard sample plot (20 m × 20 m) was set up along the gradient of the slope position (upper slope, middle slope, down slope). According Three sets of experiments were established for each slope position, and 10 plots were established for each forest stand.

### Sample collection

**Fig 2:**
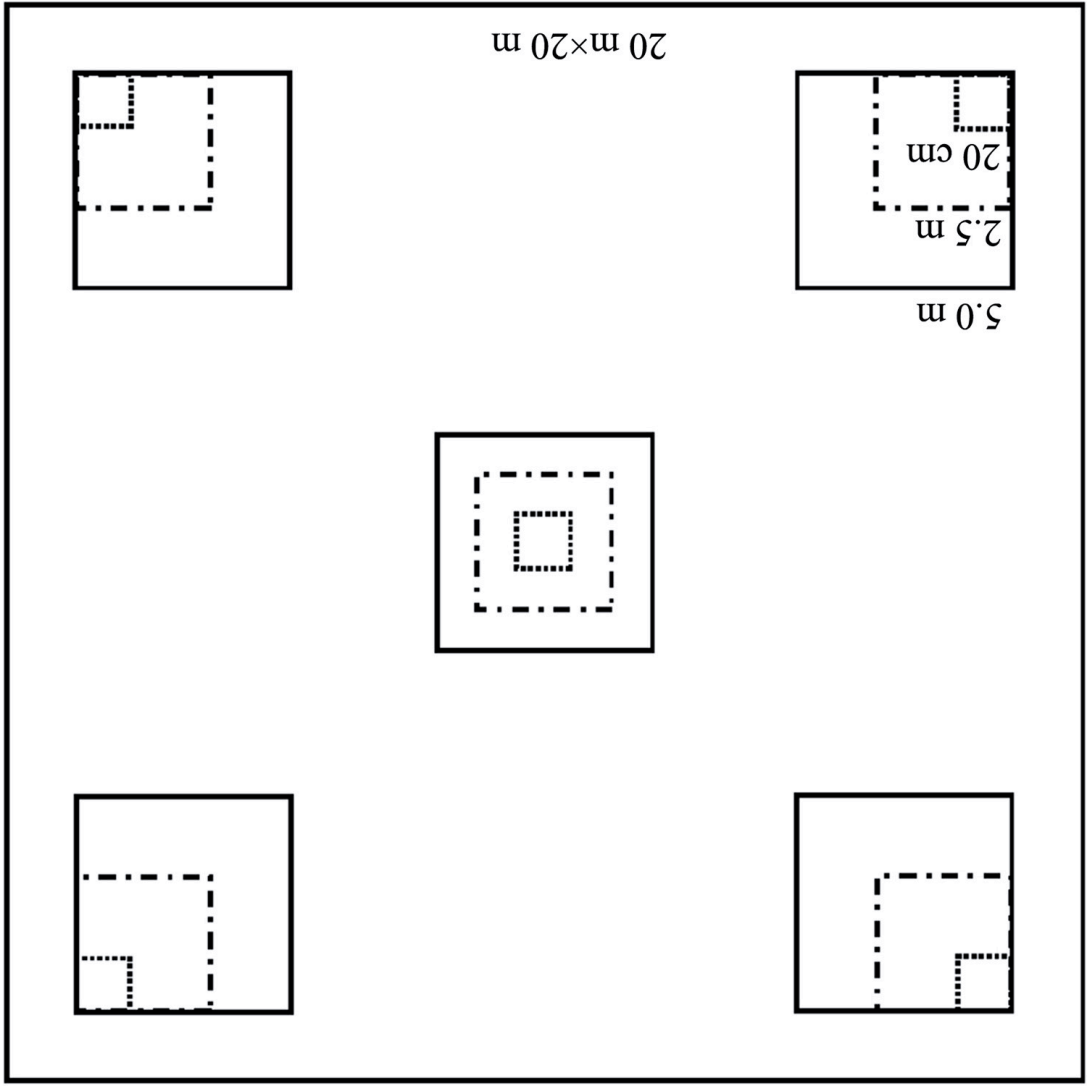
Experimental plot configuration.

Five samples were collected at equal distances along the diagonal of the plot. The types of surface litter collected and the corresponding plot sizes were as follows: 1-h time-lag combustibles (twigs, leaves, and dead weeds with diameters less than 0.64 cm), 20 cm × 20 cm; 10-h time-lag combustibles (twigs with diameters of 0.64–2.54 cm), 2.5 m × 2.5 m; and 100-h time-lag combustibles (dead branches with diameters of 2.54–7.62 cm), 5 m × 5 m. The 1-, 10-, and 100-h time-lag combustibles were collected and weighed. Part of each sample was then placed into a Ziplock bag and taken back to the laboratory, take a total of 750 samples. The experimental plot setup is shown in Fig 2.

### Biomass characterization and TGA analysis

Use an analytical balance to weigh 1g of the combustible sample after drying and crushing, and use the ANDMF-50 moisture tester to determine the moisture content of the combustible sample. Repeat the experiment for each group of combustibles for three times and take the average value as the combustible content of the sample.

Use an analytical balance to weigh 2g of the combustible sample after drying and crushing, and put it into the IKA C 600 automatic oxygen bomb calorimeter to determine the calorific value of the combustible sample. Repeat three times for each group of combustible samples and take the average value as the calorific value of the combustible sample. The ignition point was measured by TGA (Perkin Elmer STA 6000).

For TGA analysis (Perkin Elmer STA 6000), using high-purity oxygen gas (purity 99.99%) as the carrier gas, and air as the ventilating atmosphere, a biomass sample weighing approximately 10 mg was placed evenly in a platinum crucible and heated linearly from ambient temperature to 600°C under nitrogen with flow rate of 100 mL∙min^−1^. The experiments were carried out non-isothermally at five different heating rates (β) of 40°C∙min^−1^ for both processes. The experiments were carried out non-isothermally at five different heating rates (β) of 40°C∙min^−1^ for both processes. To ensure the experimental repeatability within 2% margin of error, the reaction conditions were performed for at least three times. The TGA data were used to calculate the kinetic and thermodynamic parameters. The Instrument Information is shown in Table 1.

**Table 1:** Instrument information.

Instrument	Producer	Andorigin	Measurement index
**ANDMF-50**	Android	Japan	Moisture content
**IKA C600**	IKA	Germany	Calorific value
**STA6000**	PerkinElme	America	Thermogravimetric analysis

### Coats–Redfern kinetic analysis

The biomass pyrolysis reaction can be abbreviated as A (solid) → B (solid) + C (gas). The kinetic equations are

$$\frac{d(\alpha )}{dt}=kf(\alpha )=Aexp(\frac{-E}{RT})f(\alpha ),$$ (1)

$$\alpha =\frac{\left(m_{0}-m\right)}{m_{0}-m_{\infty }},$$ (2)

and

$$k=Aexp\left(\frac{-E}{RT}\right),$$ (3)

where α is the degree of decomposition (%); *m*_*0*_ is the initial mass of the sample (g); *m*_*∞*_ is the mass of residues that cannot be decomposed (g); *k* is the Arrhenius rate constant; *E* is the reaction activation energy (kJ∙mol^−1^); *A* is the pre-referred factor; *R* is the universal gas constant (8.314 J∙mol^−1^); and *T* is the thermodynamic reaction temperature.

The pre-exponential factor was calculated using the Coats–Redfern model with a heating rate of 40°C∙min^−1^. When selecting the reaction order *n*, rate of reaction can be written as, the integral parts of Eqs (4)–(6) have no exact solution. Applying an asymptotic series and neglecting higher-order terms gives the following solutions:

$$\ln[\frac{-\ln\left(1-\alpha \right)}{T^{2}}]=\ln\left[\frac{AR}{\beta E}(1-\frac{2RT}{E})\right]-\frac{E}{RT}(for\;n=1),$$ (4)

and

$$\ln[\frac{-{\left(1-\alpha \right)}^{1-n}}{T^{2}(1-n)}]=\ln\left[\frac{AR}{\beta E}(1-\frac{2RT}{E})\right]-\frac{E}{RT}(for\;n\neq 1).$$ (5)

However, if $1-\frac{2RT}{E}\approx 1$ (1-2RT / E)≈1, the first term on the right sides of Eqs (4) and (5) is almost constant. Consider the cases where *n* equals 0.5, 0.6, 0.7, 0.8, 1.0, 1.2, and 1.5. When *n* = 1, the degree of linearization is high, and the solution becomes

$$\ln\left[\frac{g(\alpha )}{T^{2}}\right]=\ln[\frac{AR}{\beta E}]-\frac{E}{RT}.$$ (6)

$Y=\ln\left[\frac{g(\alpha )}{T^{2}}\right],\;X=\frac{1}{T},\;a=\ln\left[\frac{AR}{\beta E}\right],\;b=\frac{-E}{R},$ the kinetic equation can be simplified as

Y=a+bX. (7)

Using $\ln\left[\frac{g(\alpha )}{T^{2}}\right]$ to plot $\frac{1}{T}$, the corresponding fitting equation and the linear correlation coefficient *r* can be obtained. According to Eq (7), the sample can be obtained from the slope and intercept of the straight line *E* and *A*.

### Pyrolysis characteristic index

To more comprehensively evaluate the burning of the samples, the combustibility was comprehensively evaluated. The pyrolysis characteristic index *P* indicates the degree of difficulty of pyrolysis [34]:

$$P=\frac{\left|d_{max}\right|}{t_{max}t_{s}(t_{2}-t_{1})},$$ (8)

where |d_max_| is the maximum weight-loss rate (%∙min^−1^); *t*_*max*_ is the temperature (°C) corresponding to the maximum weight-loss rate; *t*_*s*_ is the initial decomposition temperature (°C); (*t*_*2*_*–t*_*1*_) is the temperature difference, which corresponds to the half peak of the maximum weight-loss peak width (°C).

### Ignition index

The ignition index is also used to describe the combustion characteristics of biomass. The larger the ignition index, the better the ignition performance of combustibles [35].


$$D_{i}=\frac{{DTG}_{max}}{T_{i}\times T_{b}}$$ (9)

Where *D*_*i*_ is the ignition index, *DTG*_*max*_ is the maximum weight loss rate, %·min^-1^. *T*_*i*_ is the ignition temperature, °C. *T*_*b*_ is the burnout temperature, °C.

## Results and discussion

### Physiochemical analysis

The moisture contents of combustibles in different forest types are quite different. The moisture contents of the different forest types range from 6.0%–20.0%. The BP, P-B, and QM samples had moisture contents of less than 10%, whereas the moisture contents of the LG and L-B samples were 15.26% and 10.36%, respectively (Table 2). The combustibility is negatively correlated with moisture content, and forest types with high moisture contents have relatively low combustion levels [33]. Furthermore, all dead combustibles contain low amounts of ash (< 5%). Among the forest types in this study, the ash contents were lowest in the LG (2.94%) and L-B (3.01%) groups. Ash in combustible materials retards flaming combustion and reduces the output of volatile combustibles [36]. High ash contents result in poor combustion performance and enhanced fire resistance [37]. The calorific value of a combustible material affects its ignition temperature and the rate of fire spread. High combustibility corresponds to high fire intensity [38]. In this study, the calorific value between 16.19–18.09 kJ∙g^−1^, Similar to the research results of other scholars [35, 39, 40], and the calorific value for LG (16.19 kJ∙g^−1^) was significantly lower than those of the other groups. The ignition points of the dead surface combustibles from the different forest types were approximately 270.0°C–290.0°C. Among the samples, the highest ignition point was observed for BP (283.22°C), while the lowest was found for QM (275.25°C). Combustibles with higher ignition points have higher temperature requirements for the external fire source and require longer heating times [37].

**Table 2:** Physical and chemical properties of dead combustibles.

Forest type	Moisture content (%)	Ash content (%)	Calorific value (kJ∙g^−1^)	Ignition point (°C)
**L-B**	10.36±0.0096	3.01±0.0913	17.25±1.18	278.91±9.35
**BP**	7.10±0.0087	3.88±0.0764	17.55±1.83	283.22±12.25
**LG**	15.26±0.0700	2.94±0.0360	16.19±1.72	277.08±6.70
**P-B**	7.57±0.0800	3.17±0.0423	18.09±0.75	281.05±14.38
**QM**	8.03±0.0270	4.11±0.0347	17.58±0.83	275.25±8.93

### Correlation and principal component analyses of the physical and chemical properties of combustibles

**Fig 3:**
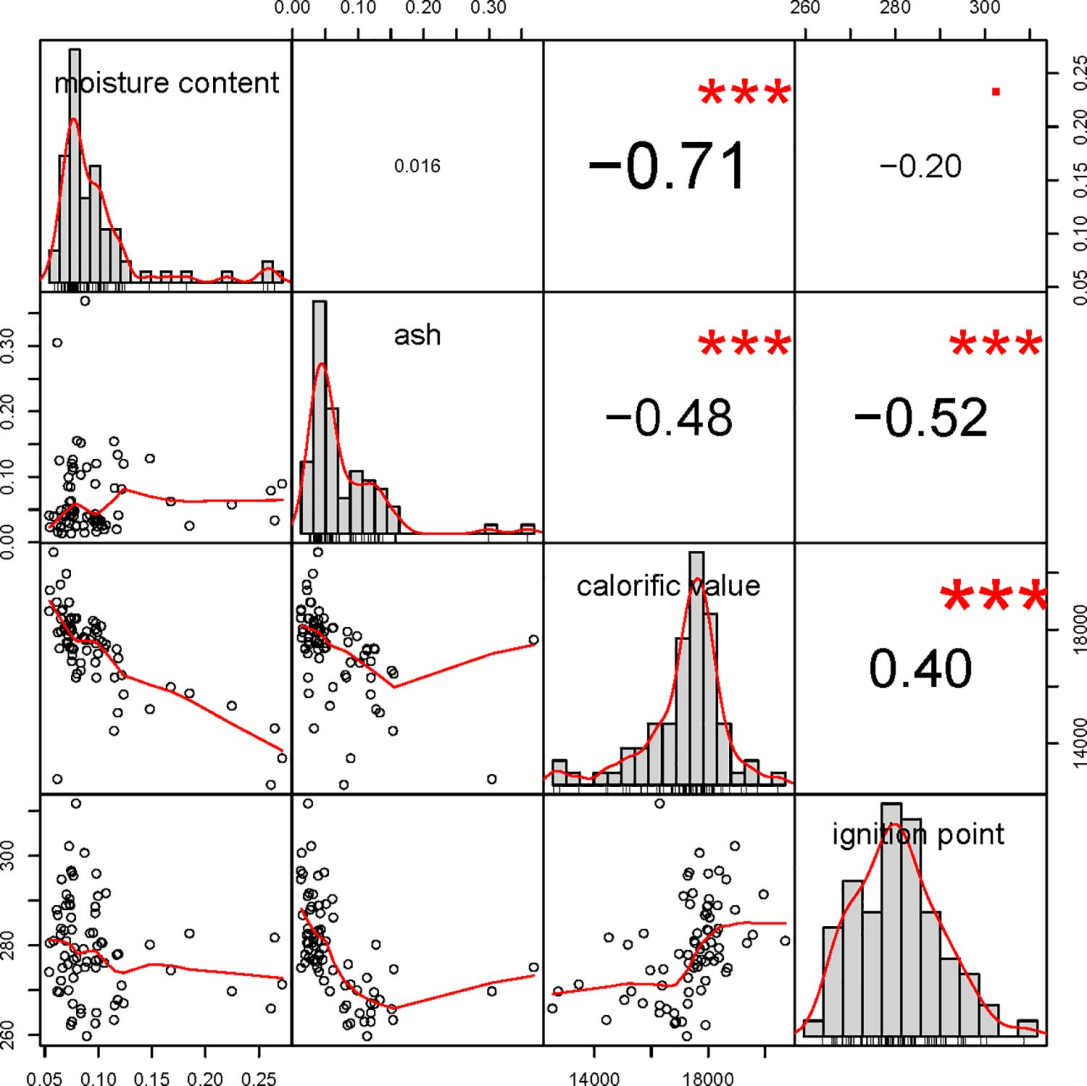
Correlation analysis of each attribute.

The physical and chemical properties of combustibles have significant effects on their combustibility [5]. The physical and chemical properties of combustibles do not exist independently; rather, they are interrelated, and their combined effect determines the combustion performance [36]. As shown in Fig 3, the combustible moisture content was significantly negatively correlated with the calorific value. This is primarily because during the combustion process, the decomposition of water requires a large amount of heat, which hinders combustion and energy accumulation. Meanwhile, the ash content was significantly negatively correlated with both calorific value and ignition point (Fig 3). Ash is primarily composed of minerals, which inhibit the release of energy during combustion. A significant positive correlation was observed between calorific value and ignition point, which can be attributed to the fact that a higher ignition point results in a longer energy accumulation time and a larger calorific value.

**Fig 4:**
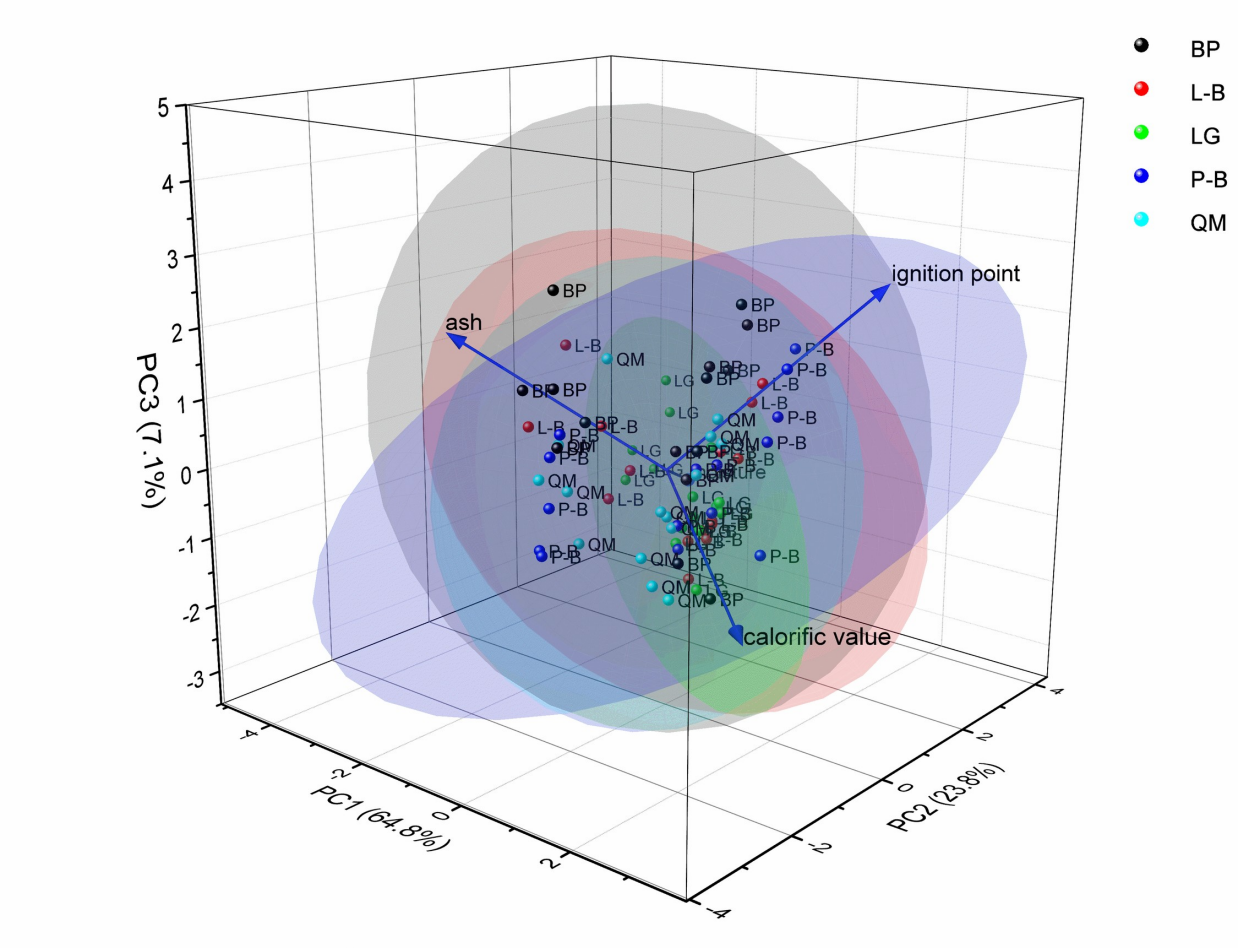
Principal component analysis of each attribute.

As shown in Fig 4, principal component 1 (PC1; 64.8%), PC2 (23.8%), and PC3 (7.1%) explain the 95.7 degree of these four variables. Calorific value and ignition point had a good correlation with PC1; thus, PC1 and these two variables are essentially oblique. PC2 and moisture were basically parallel, indicating that PC2 could represent moisture.

By sorting the comprehensive values of *Y* obtained from the principal component analysis, the combustibility of the different combustibles can be revealed. As shown in Table 3, the forest types with relatively high combustibility values and comprehensive combustibility values for surface dead fuel with 1-, 10-, and 100-h time lags were L-B, BH, and P-B. The forest types with relatively low combustibility values and comprehensive combustibility values for 1-, 10-, and 100-h time lags were L-B and LG.

**Table 3:** Comparison of comprehensive flammability indicators.

Type	1 h		10 h		100 h		Comprehensive Combustibility	Rank
	Flammability	Rank	Flammability	Rank	Flammability	Rank		
**L-B**	−4.60	4	1.51	4	1.76	4	−1.33	4
**BP**	−3.37	3	2.73	1	3.57	2	2.93	2
**LG**	−6.87	5	0.76	5	−1.15	5	−7.26	5
**P-B**	−1.89	1	1.97	2	4.63	1	4.71	1
**QM**	−2.96	2	1.60	3	2.3	3	0.94	3

### Analysis of combustible material load

According to the degree of recovery in the moisture content, dead combustibles can be divided into 1-, 10-, 100-, and 1000-h time-lag combustibles [36]. In this study, the combustible load was determined as the sum of the combustible loads of the 1-, 10-, and 100-h time-lag combustibles. Table 4 shows the dead fuel loads on the surfaces of different forest types. The total dead combustible load of the five forest types was 247.096 kg·m^−2^. Most of the combustible load was contributed by the 1-h time-lag combustibles (239.059 kg·m^−2^ or 96.75% of the total load). 1-h combustible load accounted for a relatively large proportion of the total surface dead combustible load. Therefore the litter on the ground under the forest should be cleaned up in a timely manner.

**Table 4:** Dead fuel load on the forest surface (kg·m^−2^).

Type	1-h time-lag combustibles	10-h time-lag combustibles	100-h time-lag combustibles	Total combustibles
**L-B**	46.941±0.312	0.947±0.011	0.747±0.010	48.635±0.314
**BP**	48.188±0.364	0.722±0.009	0.488±0.007	49.399±0.369
**LG**	53.922±0.349	1.122±0.012	0.582±0.008	55.626±0.349
**P-B**	52.908±0.444	0.906±0.008	0.467±0.005	54.281±0.443
**QM**	37.099±0.210	1.430±0.018	0.625±0.008	39.155±0.212
**Total**	239.059±0.362	5.129±0.013	2.908±0.008	247.096±0.362

The 1-h time-lag combustible load decreased in the following order among the different forest types: LG > P-B > BP > L-B >QM. The pine needles in LG have a strong capacity for water absorption, which leads to a slow rate of water loss. Since the temperature in the forest is low, the combustibles decompose slowly, and the long-term accumulation of combustibles results in a relatively large time lag for LG [3]. In contrast, L-B and QM contain more shrubs and grasses. As the canopy density of the forest increases, the humidity increases, which speeds the decomposition of surface litter; thus, the 1-h time-lag fuel loads were relatively small in these forest types.

The 10-h time-lag combustible load decreased in the following order among the different forest types: QM > LG > L-B > P-B > BP. In QM and LG, the diameters and surface areas of branches are smaller than those of leaves and weeds, resulting in slow water loss, slow combustible decomposition, and a relatively large load of combustibles with a 10-h time lag [37]. However, BP is in the pioneer stage of succession of LG. As the larch forest grows, the canopy becomes more closed, and growth is gradually inhibited. Therefore, the 10-h time-lag combustible load is relatively low in L-B.

The 100-h time-lag combustible load decreased in the following order among the different forest types: L-B > QM > LG > BP > P-B. L-B is naturally sparse and contains numerous dead branches, leading to a relatively large l00-h time-lag combustible load in this forest type. The five forest types showed different ignition temperatures of combustible materials with a time lag of 1 h, and these temperatures were lower than those of combustible materials with time lags of 10 and 100 h. Among the forest types, the flammability of dead combustibles with 1-, 10-, and 100-h time lags was relatively high in QM and BP and relatively low in L-B, QM, and P-B.

### Thermal decomposition of combustibles

**Fig 5:**
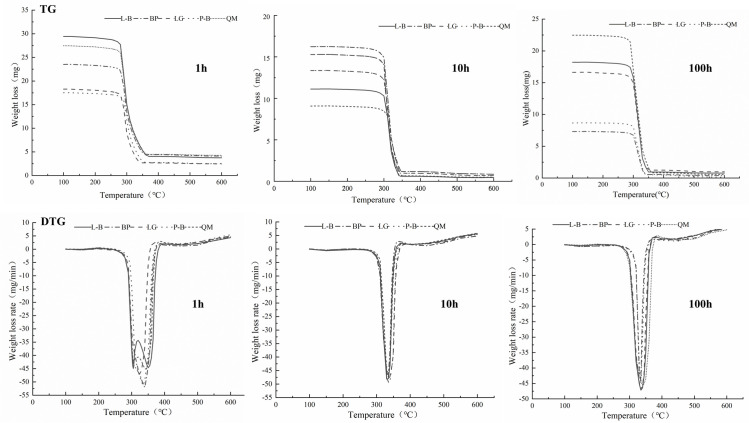
TG and DTG curves of combustibles with 1, 10, 100h time lag in different forest types.

The trends in the TGA and derivative thermogravimetry (DTG) curves obtained under oxidizing conditions were different from the trends observed in the pyrolysis curves. The DTG curve of the combustible sample showed a small weight-loss peak that might be attributed to the diffusion of oxygen into biomass particles [41]. This zone is referred to as a burning region in which volatiles are released and burned [42]. The results of the present study confirmed that the pyrolysis process for the five forest types can be divided into three stages: dehydration, rapid pyrolysis, and carbonization (Fig 5).

At temperatures below 230°C, the weight-loss rates of combustibles were 2.30%–3.60%, 0.66%–1.18%, and 0.41%–1.33% for 1-, 10-, and 100-h time lags. When the 1-h time-lag combustible samples reached the rapid pyrolysis stage, the TGA curve dropped sharply, and a weightless step appeared. The temperature range of weight loss was 264°C–390°C, and the weight-loss rate reached 82.26%. Among the samples, the weight-loss rate was largest for L-B followed by LG and B-S. For the 10-h time-lag combustibles, the weight loss interval in the rapid pyrolysis stage was 280°C–411°C, and the weight-loss rate reached 90.83%. Among the forest types, the largest weight-loss rate was observed in B-S followed by L-B and QM. For the 100-h time-lag combustible samples in the rapid pyrolysis stage, the weight-loss interval was 267.5°C–408°C, and the weight-loss rate reached 94.81%. Among the samples, the highest weight-loss rate appeared in QM followed by L-B and P-B.

The 1-, 10-, and 100-h time-lag combustibles began to slowly pyrolyze and gradually carbonize when the temperature exceeded 390°C, 408°C, and 411°C, respectively. The TGA and DTG curves gradually became stable with increasing temperature, after which the weight loss of the sample remained basically unchanged. At this stage, only a small amount of unpyrolyzed residual materials were being slowly pyrolyzed, and the rest was attributed to a small amount of incompletely decomposed solid coke and non-decomposable ash [43]. Based on the above results, the dead combustible materials with 1-, 10-, and 100-h time lags show relatively high thermal stability in the LQ and QM forest types. In contrast, the thermal stability is relatively low for the L-B and P-B forest types.

The ignition temperature, which is the lowest temperature required for combustibles to burn continuously, can be used to characterize the thermal stability of combustibles [44]. The tangent method was used to determine the ignition and burnout temperatures of the samples. Among the time delays, the ignition temperature was lowest for the 1-h time delay and highest for the 100-h time delay (Table 5). For the 1-h time-lag combustible samples, the most flammable were those from the QM forest type, while the least flammable were from the P-B forest type. The same trend was observed for the 100-h time-lag combustible samples. In contrast, for the 10-h time-lag combustibles, the highest flammability was observed for the LG forest type, while the lowest flammability was found in the QM forest type. As the heating rate increased in the different forest types, the weight-loss rate increased to different degrees; the smallest increase in weight-loss rate was found in the LG forest type. These results indicate that the heating rate had little effect on the weight-loss rate of the combustibles.

**Table 5:** Ignition and burnout temperatures (°C) of combustible samples with different time lags.

Type	1 h		10 h		100 h	
	Ignition temperature	Burnout temperature	Ignition temperature	Burnout temperature	Ignition temperature	Burnout temperature
**L-B**	263.3±4.48	369.4±8.56	287.1±6.04	344.8±5.32	276.5±7.45	350.5±6.56
**BP**	265.8±3.38	355.6±6.33	281.0±4.70	339.3±4.90	295.5±12.47	336.3±8.72
**LG**	265.9±5.63	341.4±6.28	282.7±5.52	350.2±7.82	278.1±2.56	348.5±7.88
**P-B**	269.5±3.92	356.5±4.56	283.7±3.91	353.2±7.56	296.6±4.37	338.0±5.36
**QM**	263.0±4.86	361.8±7.52	288.9±5.90	346.0±9.22	275.0±7.35	364.4±7.80

### Kinetic analysis

The value of *E* represents the minimum energy required for the reaction to begin. That is, a high *E* value indicates that it is difficult for the reaction to start, and a low *E* value corresponds to a fast reaction rate [12]. In general, *E* increases with increasing temperature due to parallel reaction routes, each with a different *E* value [45]. The activation energies, pre-exponential factors, and reaction models were obtained for the equivalent single-step pyrolysis reaction model. The kinetic parameters of the forest fuel samples were obtained with the commonly used Coats–Redfern method [33]. The kinetic parameters obtained herein were consistent with previously reported values.

Based on the correlation coefficient *R*^2^ (Table 6), the first-order fitting equation resulted in a good linear relationship, indicating that the Coats–Redfern integral method is suitable for describing the pyrolysis behavior of forest combustible materials in an oxygen-containing atmosphere. The *R*^2^ value for the correlation between the activation energy *E* and the frequency factor *A* is related to the rate of the pyrolysis reaction. The magnitude of the activation energy *E* reflects the difficulty of the pyrolysis process and the pyrolysis characteristics of the reactants. The higher the activation energy, the more energy required for the reaction and the more difficult it is for the reaction to proceed. As shown in Table 5, in the rapid pyrolysis stage, the activation energy of 1-h time-lag combustibles ranged from 22.76–42.97 kJ·mol^−1^, and among the forest types, *E* tended to be highest in LG. The activation energy of 10-h time-lag combustibles ranged from 58.26–72.33 kJ·mol^−1^, and *E* was highest in the QM forest type. For 100-h time-lag combustibles, *E* ranged from 8.93–59.15 kJ·mol^−1^, and the *E* values were highest in the LG forest type. Based on the above results, the LG, and QM forest types had relatively high combustibility values and comprehensive combustibility values for 1-, 10-, and 100-h time lags. Meanwhile, the P-B forest type had relatively low combustibility values and comprehensive combustibility values.

**Table 6:** Pyrolysis parameters and pyrolysis characteristic indices of fuel samples with different time lags.

Type	Time lag	Temperature range (°C)	Fitting equation	*E* (kJ·mol^−1^)	*A* (s^−1^)	*R* ^2^	*P* (10^−6^ °C∙min^−1^)	*D*_*i*_ (×10^−5^)
**L-B**	1 h	283.1~369.4	*Y* = −2736.98*X*−2.74	22.76	8.87×10^17^	0.86	4.86	4.62
	10 h	287.1~344.8	*Y* = −8649.74*X*+15.29	71.92	1.33×10^−60^	0.95	3.21	4.86
	100h	276.5~350.5	*Y* = −7067.39*X*+10.49	58.76	8.07×10^−40^	0.93	6.21	4.87
**BP**	1 h	265.8~355.6	*Y* = −4483.98*X*+2.65	37.28	5.78×10^−6^	0.92	5.54	5.50
	10 h	281.0~339.3	*Y* = −8623.43*X*+15.49	71.69	1.82×10^−61^	0.95	4.92	4.97
	100h	295.5~336.3	*Y* = −10898.95*X*−22.05	9.06	7.85×10^−91^	0.93	7.12	4.44
**LG**	1 h	265.9~341.4	*Y* = −5167.76*X*+5.22	42.97	4.51×10^−17^	0.94	6.66	5.20
	10 h	282.7~350.2	*Y* = −7008.56*X*+9.99	58.26	1.13×10^−37^	0.95	6.33	5.00
	100h	278.1~348.5	*Y* = −7114.711*X*+10.62	59.15	2.15×10^−40^	0.94	7.00	4.85
**P-B**	1 h	269.5~356.5	*Y* = −4920.22*X*+3.86	40.90	3.46×10^−11^	0.94	7.80	5.34
	10 h	283.7~353.2	*Y* = −7440.34*X*+11.39	61.86	1.06×10^−43^	0.95	6.19	4.85
	100h	296.6~338.0	*Y* = −10737.36*X*+23.39	8.93	5.20×10^−88^	0.95	7.62	4.49
**QM**	1 h	263.0~361.8	*Y* = −4219.01*X*+1.76	35.08	0.03×10^4^	0.91	6.29	4.74
	10 h	288.9~346.0	*Y* = −8700.18*X*+15.21	72.33	2.94×10^−60^	0.96	7.40	4.65
	100h	275.0~364.4	*Y* = −5700.73*X*+5.99	47.40	2.23×10^−20^	0.90	5.13	4.54

The larger the pyrolysis characteristic index *P*, the easier the pyrolysis of the sample [46]. According to the obtained *P* values, the pyrolysis of dead surface fuels with 1-, 10-, and 100-h time lags is relatively difficult in the L-B and QM forest types. In contrasts, pyrolysis of dead combustibles is relatively easy in the P-B forest types.

Through the analysis of the ignition index, it can be seen that the ignition index of L-B and QM is relatively low, their thermal stability is relatively good, and it is difficult to decompose. On the contrary, BP and P-B have higher ignition index, and their thermal stability is poor, and they are easier to decompose.

## Conclusion

Understanding the kinetic properties of forest surface litter fuels is important for predicting the propagation of wildfire on the forest surface. A single-step pyrolysis model appears to be sufficient to estimate some features of forest fires, including the rate of fire spread and time to ignition. Such a model can be easily implemented using existing physics-based computational methods. Through comprehensive analysis, tree species such as LG, LB, and QM have the best fire resistance which can be considered as the fire-resistant tree species in the Daxing’an Mountains of Inner Mongolia to prevent the spread of forest fires. At the same time, based on the utilization of biomass energy, the thermal stability of BP and P-B tree species is relatively poor, the pyrolysis of combustibles is relatively easy, and their calorific value is relatively high, which can be used as biomass energy in the future.

## Supporting information

S1 Table Combustible load date. (XLSX)

S2 Table cor date. (CSV)

S3 Table pca date. (CSV)

S4 Table Time lag combustible physical date. (XLSX)

S5 Table Weight loss 1h date. (XLSX)

S6 Table Weight loss 10h date. (XLSX)

S7 Table Weight loss 100h date. (XLSX)

S8 Table Weight loss rate 1h date. (XLSX)

S9 Table Weight loss rate 10h date. (XLSX)

S10 Table Weight loss rate 100h date. (XLSX)
